# Vital Statistics

**Published:** 1920-08-21

**Authors:** 


### Vital Statistics.

The vital statistics for the June quarter show an additional population of 130,000, and a satisfactory feature is the decrease in infantile mortality, which is 82 per 1,000, or 6 per 1,000 below the average for the ten preceding years. Illegitimate births increased by 2,200 in actual figures, but not in proportion to the total births. Roughly, there was one illegitimate in every twenty births, compared with one in every fifteen last year. *The Times*, in an analysis of the statistics, says the excess of male births over female births—in the proportion of 1,045 to 1,000—is well within the range of what may be called normal variation, and insufficient to support popular beliefs in the effect of war on the determination of sex. An interesting table of the birth and death rates of certain Continental cities for the second quarter of the year seems to show that economic conditions are not pressing on Germany with great severity. The death-rate for London was 12.5 per 1,000 of the population (calculated as for the whole year), for Antwerp 9.3, Amsterdam 9.7, Stockholm 12.5, Christiania 11.6, Berlin 13.2, Hamburg 12.6, Leipzig and Dresden 12.3, Cologne 14, and Frankfort 10.4. The London birth-rate, similarly reduced to an annual rate, but including only civilians, was 28.3, that for Antwerp 21.9, Amsterdam 23.9, Stockholm 18.6, Christiania 22.7, Berlin 17.[illegible], Hamburg 22.1, Leipzig, 23.1, Dresden 22.[illegible], Cologne 26.9, and Frankfort 20.4. On the other hand, the figures for Vienna and Prague show lower birth-rates and higher death-rates.

